# Successful management of hemodynamic instability secondary to saddle pulmonary embolism-induced cardiac arrest using VA-ECMO in advanced malignancy with brain metastases

**DOI:** 10.1186/s13019-022-02044-w

**Published:** 2022-12-05

**Authors:** Jianneng Pan, Xiaoyang Zhou, Zhaojun Xu, Bixin Chen

**Affiliations:** Department of Intensive Care Medicine, HwaMei Hospital, University of Chinese Academy of Sciences, Ningbo, 315000 Zhejiang China

**Keywords:** Saddle pulmonary embolism, Hemodynamic instability, Cardiac arrest, VA-ECMO, Malignancy, Metastases

## Abstract

**Background:**

Saddle pulmonary embolism (SPE) represents a rare type of venous thromboembolism that frequently causes circulation collapse and sudden death. While venoarterial extracorporeal membrane oxygenation (VA-ECMO) has been well established as a salvage treatment for SPE-induced circulatory shock, it is infrequently administered in patients with advanced malignancy, especially those with brain metastases, given the potential bleeding complications and an uncertain prognosis. As far, there are rare case reports regarding the successful management of hemodynamic instability secondary to SPE-induced cardiac arrest using VA-ECMO in advanced malignancy patients with brain metastases.

**Case presentation:**

A 65-year-old woman presenting with cough and waist discomfort who had a history of lung cancer with brain metastases was admitted to the hospital to receive chemoradiotherapy. She suffered sudden cardiac arrest during hospitalization and returned to spontaneous circulation after receiving a 10-min high-quality cardiopulmonary resuscitation. Pulmonary embolism was suspected due to the collapsed hemodynamics and a distended right ventricle identified by echocardiography. Subsequent computed tomographic pulmonary angiography revealed a massive saddle thrombus straddling the bifurcation of the pulmonary trunk. VA-ECMO with adjusted-dose systemic heparinization was initiated to rescue the unstable hemodynamics despite receiving thrombolytic therapy with alteplase. Immediately afterward, the hemodynamic status of the patient stabilized rapidly. VA-ECMO was successfully discontinued within 72 h of initiation without any clotting or bleeding complications. She was weaned off invasive mechanical ventilation on the 6th day of intensive care unit (ICU) admission and discharged from the ICU 3 days later with good neurological function.

**Conclusion:**

VA-ECMO may be a ‘bridging’ therapy to circulation recovery during reperfusion therapy for SPE-induced hemodynamic collapse in malignancy patients with brain metastases.

## Introduction

Saddle pulmonary embolism (SPE) represents a rare type of venous thromboembolism that occurs in approximately 2.6–5.4% of all acute pulmonary embolism (PE) cases and frequently leads to hemodynamic collapse and sudden death [1–4]. As conventional reperfusion therapy including thrombolysis and thrombectomy is less efficacious in mitigating hemodynamic instability, venoarterial extracorporeal membrane oxygenation (VA-ECMO) has increasingly emerged as a salvage therapy for PE-induced hemodynamic instability, which is typically defined as persistent hypotension, obstructive shock, or cardiac arrest [5]. Despite this, the appropriate selection of patients eligible for VA-ECMO remains an ongoing challenge in clinical practice. This is especially true for advanced malignancy patients with brain metastases given the potential bleeding complications and uncertain prognosis [6, 7]. Immunosuppression and irreversible central nervous system pathology have historically been considered to be relatively contraindicated to the application of extracorporeal membrane oxygenation (ECMO) [8]. Consequently, despite a largely increased number of ECMO runs over the past decade, the ECMO runs for neoplasms remain quite infrequent [9]. Herein, we reported a rare case describing the successful management of hemodynamic deterioration secondary to SPE-induced cardiac arrest using VA-ECMO in a lung cancer patient with brain metastases.

## Case presentation

A 65-year-old woman presenting with cough and waist discomfort who had a history of lung adenocarcinoma with multiple metastases to regional lymph nodes, bones, the left adrenal, and the brain was admitted to our hospital to receive chemoradiotherapy. On admission, her vital signs were normal, and supplemental oxygen therapy was not required. During hospitalization, she received one dose of pemetrexed (0.8 g) and carboplatin (500 mg) for chemotherapy. Six days later, she received local radiotherapy on the lumbar spine a total of 6 times. According to the Padua prediction score [10], she was judged as having a low risk of venous thromboembolism (VTE) and did not receive prophylactic anticoagulation for VTE. On the day after terminating radiotherapy, she suffered a sudden cardiac arrest on her way to the bathroom. Cardiopulmonary resuscitation (CPR) was performed immediately. Fortunately, she returned to spontaneous circulation after receiving a 10-min high-quality CPR. She was intubated and transferred to the intensive care unit (ICU).

**Fig. 1:**
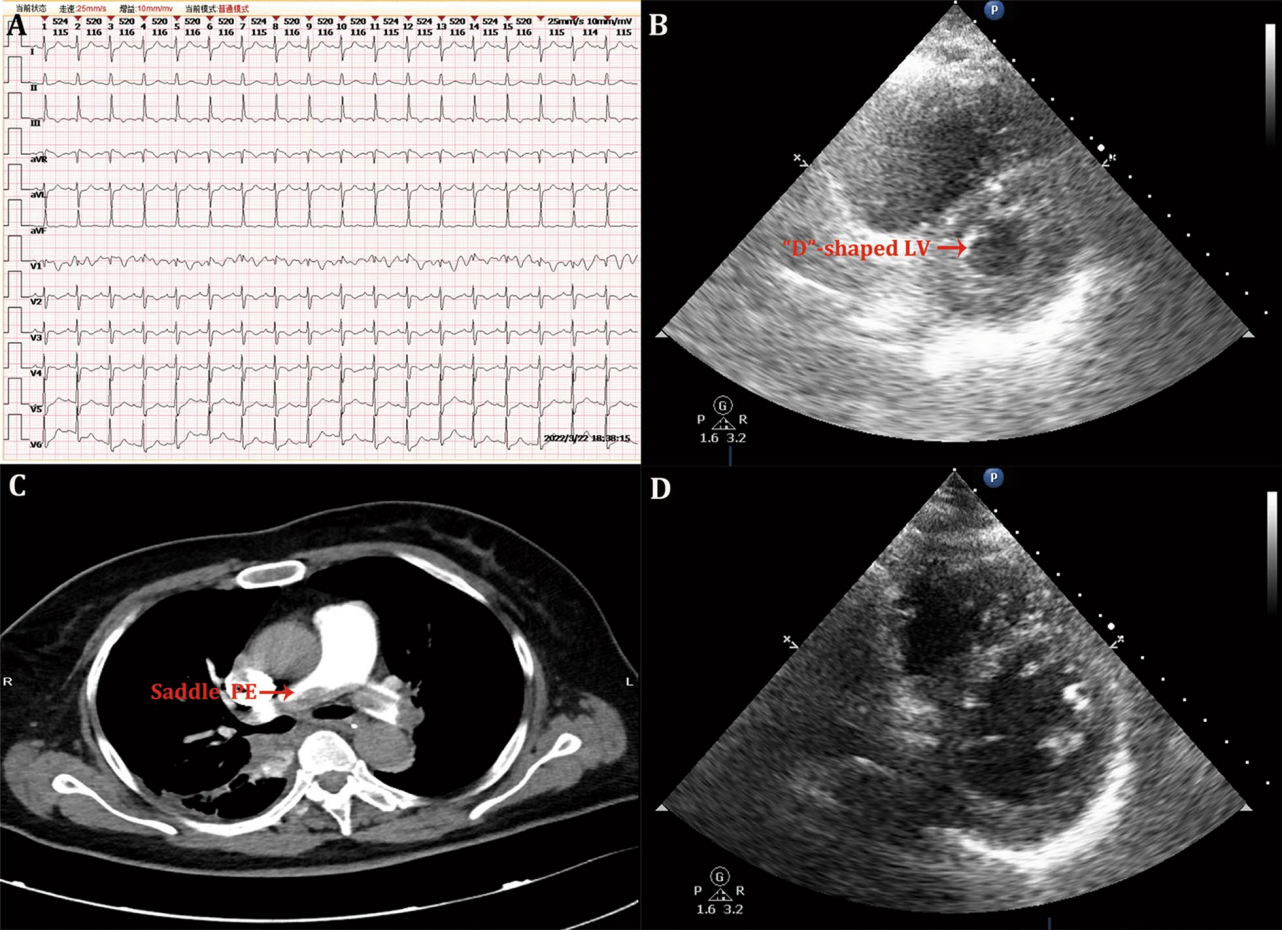
Clinical examinations supporting the diagnosis of saddle pulmonary embolism. **A** Electrocardiogram indicating S_I_ Q_III_ T_III_ and negative T wave in V1–4 leads; **B** ultrasound cardiography showing an enlarged right ventricle and a flattened intraventricular septum during systole; **C** computed tomographic pulmonary angiography revealing a saddle thrombus straddling the bifurcation of the pulmonary trunk; **D** ultrasound cardiography on ECMO day 3. PE pulmonary embolism; LV left ventricle; ECMO extracorporeal membrane oxygenation

Upon admission to the ICU, she regained consciousness, but the collapsed hemodynamics persisted, with a mean arterial pressure (MAP) of 62 mmHg on a regime of norepinephrine (NE) of 0.6 μg/kg/min. The arterial blood gas test revealed a high level of lactate of 8.5 mmol/L and partial pressure of oxygen of 110 mmHg under invasive mechanical ventilation (IMV) with a fraction of inspired oxygen of 50%. An electrocardiogram showed sinus tachycardia and S_I_ Q_III_ T_III_, with negative T wave in V1–4 leads (Fig. 1A). An ultrasound cardiography showed an enlarged right ventricle and a flattened intraventricular septum (“D”-shaped left ventricle) during systole (Fig. 1B). The blood test revealed a high level of D-Dimer of 37,198 ng/mL. Based on these findings, PE was suspected to be the cause of cardiac arrest. Computed tomographic pulmonary angiography was subsequently performed and revealed a massive saddle thrombus straddling the bifurcation of the pulmonary trunk and extending into the left and right pulmonary arteries (Fig. 1C), suggesting the diagnosis of SPE. Unfortunately, the cardiothoracic and vascular surgery team in our hospital could not provide mature embolectomy or percutaneous catheter-directed treatment. Thus, intravenous thrombolysis was considered, and a half-dose regimen of recombinant tissue-type plasminogen activator (rtPA, 50 mg of alteplase, Boehringer Ingelheim, Germany) was administrated for 120 min given the high risk of bleeding.

However, the patient’s condition was not improved and deteriorated on the following morning; she presented with anuria for more than 6 h; the MAP decreased to 51 mm Hg with the infused dose of NE increasing to 0.9 μg/kg/min, and the lactate level progressively increased to 11.0 mmol/L. The decision on the initiation of VA-ECMO was rapidly made by the ECMO team in the ICU. The percutaneous cannulation was performed under ultrasound guidance, with a 21-Fr drainage catheter (Maquet Cardiopulmonary AG, Rastatt, Germany) placing at from the right femoral vein to the right atrium and a 15-Fr arterial catheter (Maquet Cardiopulmonary AG, Rastatt, Germany) inserting into the left femoral artery. Additionally, a 6-Fr introducer sheath (Cordis, USA) was inserted into the left superficial femoral artery to prevent distal limb ischemia. The VA-ECMO pump was initiated at 3000 rpm, resulting in a blood flow of 3.1 L/min. Meanwhile, continuous renal replacement therapy was also started, with the dialysis catheter connecting to the ECMO circuit. Systemic anticoagulation with adjusted-dose unfractionated heparin was implemented to prevent thrombotic events, with an initial dose of 5.6 U/kg/h (the patient body weight of 67 kg) without a loading dose, maintaining an activated coagulation time (ACT) range of 150–170 s and an activated partial thromboplastin time (aPTT) of 40–60 s to avoid intracranial hemorrhage (ICH). Given the narrow target range of ACT and aPTT, the dose of unfractionated heparin for each adjustment was between 0.9 and 1.9 U/kg/h. To closely monitor the coagulation function, we adopted a frequent examination of ACT and aPTT which were repeated every 2 h and 4 h, respectively.

After commencing VA-ECMO, her hemodynamic status stabilized rapidly; the MAP increased to 80 mmHg, accompanied by a progressively decreased infused dose of NE. The blood lactate level was normalized within 12 h and the use of NE was discontinued within 48 h of VA-ECMO initiation. On the 3rd day of VA-ECMO beginning, ultrasound cardiography was reevaluated and showed that the enlarged right ventricle was mitigated and the “D”-shaped left ventricle disappeared during the cardiac cycle (Fig. 1D). Thus, she started to be weaned off VA-ECMO, and VA-ECMO was successfully ceased within 72 h of initiation without any clotting or bleeding complications. The patient was weaned off IMV on the 6th day of ICU admission and transferred from the ICU to the general ward 3 days later with good neurological function. One week later, renal recovery (no requirement of renal replacement therapy) was achieved. Finally, the patient was discharged from the hospital 2 weeks later after ICU discharge. At a follow-up of 5 months after discharging from the ICU, the patient is still alive and has a good quality of life.

## Discussion and conclusion

In this case report, we described our experience in the successful management of hemodynamic deterioration secondary to SPE-induced cardiac arrest using VA-ECMO with adjusted-dose systemic anticoagulation in a lung cancer patient with brain metastases. The patient had no clotting or bleeding complications during VA-ECMO treatment and was discharged from the hospital with good neurological function. This case suggests the effectiveness of VA-ECMO as a salvage therapy for SPE-induced hemodynamic collapse in malignancy patients with brain metastases.

To our knowledge, there is a scarcity of case reports on the application of VA-ECMO to successfully rescue SPE-induced hemodynamic collapse in advanced malignancy patients with brain metastases. Indeed, ECMO is infrequently applied in the cohort of patients with advanced malignancy, especially those with brain metastases. According to the latest report from the extracorporeal life support organization registry, the percentage of ECMO runs for neoplasms is around 3% of all ECMO runs, and ECMO runs for the central nervous system neoplasms account for only 1.3% of all ECMO runs for neoplasms [9]. The extremely reduced utilization of ECMO in the population of advanced malignancy with brain metastases might attribute to the following potential reasons: first, the long-term prognosis of a patient with advanced malignancy receiving ECMO may be undesirable; second, the potential ECMO-related bleeding complications were frequent and, sometimes, lethal [11, 12]. Hence, when a malignancy patient with brain metastases developed respiratory failure or circulatory shock, his/her relatives or physicians in charge might abandon the option of ECMO treatment in consideration of the high cost and uncertain prognosis. Cost-effectiveness has always been an important factor that relatives or physicians have to consider before establishing an ECMO circuit. Several studies reported that VA-ECMO was a cost-effective treatment for cardiogenic shock, cardiac arrest, or cardiotoxicity poisonings [13–16]. However, there was no study investigating the cost-effectiveness of VA-ECMO in the special subgroup population (i.e., malignancy) so far. Despite this, we believe that VA-ECMO remains a cost-effective treatment for PE-induced refractory shock in patients with malignancy, provided that strict indications and contraindications are applied while respecting the relatives' willingness to rescue. Based on the experience of our ECMO center, we are still willing to discuss with the relatives the possibility of applying ECMO to treat acute hemodynamic instability in malignancy patients, as long as their basic nutritional status is good and has not progressed to cachexia.

Despite the above-mentioned adverse factors, VA-ECMO seems to manifest expected survival benefits in the treatment of massive PE-induced circulatory instability. Most recently, a retrospective large-scale study suggested that compared to thrombolysis alone, VA-ECMO treatment alone or as part of conventional reperfusion therapy offered survival benefits in patients with PE deteriorating to cardiac arrest [17]. Regarding the reperfusion therapy for acute PE in malignancy patients with brain metastases, surgical pulmonary embolectomy or percutaneous catheter-directed treatment is preferred to thrombolysis due to the high risk of bleeding [5]. However, the patient in this case report received a half-dose of rtPA (50 mg over 2 h) for reperfusion therapy because our hospital could not provide mature embolectomy or percutaneous catheter-directed treatment. The efficacy and safety of a half-dose regimen of rtPA were confirmed in a multicenter randomized controlled trial [18], which suggested that a half-dose regimen of rtPA, compared with the usual dose (100 mg), exhibited similar efficacy and perhaps better safety (less bleeding) in patients with acute PE. Finally, the patient in our case report was successfully managed with VA-ECMO for SPE-induced hemodynamic instability and was alive at discharge from the hospital with good neurological function, without any bleeding complications. This case report provides an important clinical implication that a half-dose regimen of rtPA combined with VA-ECMO may be a salvage therapy that is worthy of consideration for advanced malignancy patients with brain metastases who suffered severe SPE-induced circulatory shock, particularly in those hospitals that cannot provide embolectomy or thrombectomy. However, it should be recognized that this report only includes a single case, which represents a primary limitation, thus the results should be interpreted with caution in clinical practice.

Thrombotic events and bleeding complications during ECMO runs are two major contradictory issues encountered by clinicians. Systemic anticoagulation with unfractionated heparin, the most frequently used anticoagulant [19], will be inevitability accompanied by an increased risk of bleeding. A recent systematic review and meta-analysis summarized that bleeding complications occurred in 8–100% of the included patients and neurological complications (including neurological bleeding) in 8–76% of the included patients [20]. It is thus indispensable to adjust the infused dose of unfractionated heparin based on individualization. A previous study demonstrated the safety of a low-dose anticoagulation strategy including the maintenance of aPTT of 40–60 s during ECMO therapy for acute respiratory distress syndrome [21]. Therefore, we adjusted the dose of unfractionated heparin to maintain a targeted ACT range of 150–170 s and an aPTT of 40–60 s to avoid ICH given the brain metastases. Finally, the patient had no clotting or bleeding complications during VA-ECMO treatment.

In conclusion, VA-ECMO may be an effective ‘bridging’ therapy to circulation recovery during conventional reperfusion therapy for SPE-induced hemodynamic collapse in malignancy patients with brain metastases. Given the high risk of bleeding in this population cohort, an individualized anticoagulation regimen is suggested.

## Data Availability

Data sharing not applicable to this article as no datasets were generated or analyzed during the current study.

## Abbreviations

SPE: Saddle pulmonary embolism
PE: Pulmonary embolism
VA-ECMO: Venoarterial extracorporeal membrane oxygenation
ECMO: Extracorporeal membrane oxygenation
CPR: Cardiopulmonary resuscitation
ICU: Intensive care unit
MAP: Mean arterial pressure
NE: Norepinephrine
IMV: Invasive mechanical ventilation
ACT: Activated coagulation time
aPTT: Activated partial thromboplastin time
ICH: Intracranial hemorrhage
